# Cross-dehydrogenative N–N couplings

**DOI:** 10.1039/d1sc03851f

**Published:** 2021-10-19

**Authors:** Alexis Tabey, Pooja Y. Vemuri, Frederic W. Patureau

**Affiliations:** Institute of Organic Chemistry, RWTH Aachen University Landoltweg 1 52074 Aachen Germany Frederic.Patureau@rwth-aachen.de

## Abstract

The relatively high electronegativity of nitrogen makes N–N bond forming cross-coupling reactions particularly difficult, especially in an intermolecular fashion. The challenge increases even further when considering the case of dehydrogenative N–N coupling reactions, which are advantageous in terms of step and atom economy, but introduce the problem of the oxidant in order to become thermodynamically feasible. Indeed, the oxidizing system must be designed to activate the target N–H bonds, while at the same time avoid undesired N–N homocoupling as well as C–N and C–C coupled side products. Thus, preciously few intermolecular hetero N–N cross-dehydrogenative couplings exist, in spite of the central importance of N–N bonds in organic chemistry. This review aims at analyzing these few rare cases and provides a perspective for future developments.

## Abstract 2

For more than a century, the dehydrogenative formation of N–N bonds has remained mostly confidential. Several cross-dehydrogenative N–N coupling methods have appeared recently, promising a soon to come broad applicability of the concept.

## Graphical abstract

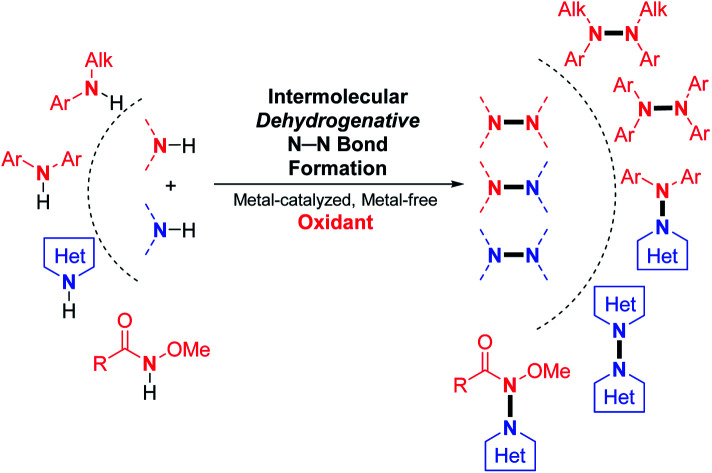

## 1. Introduction

**Scheme 1:**
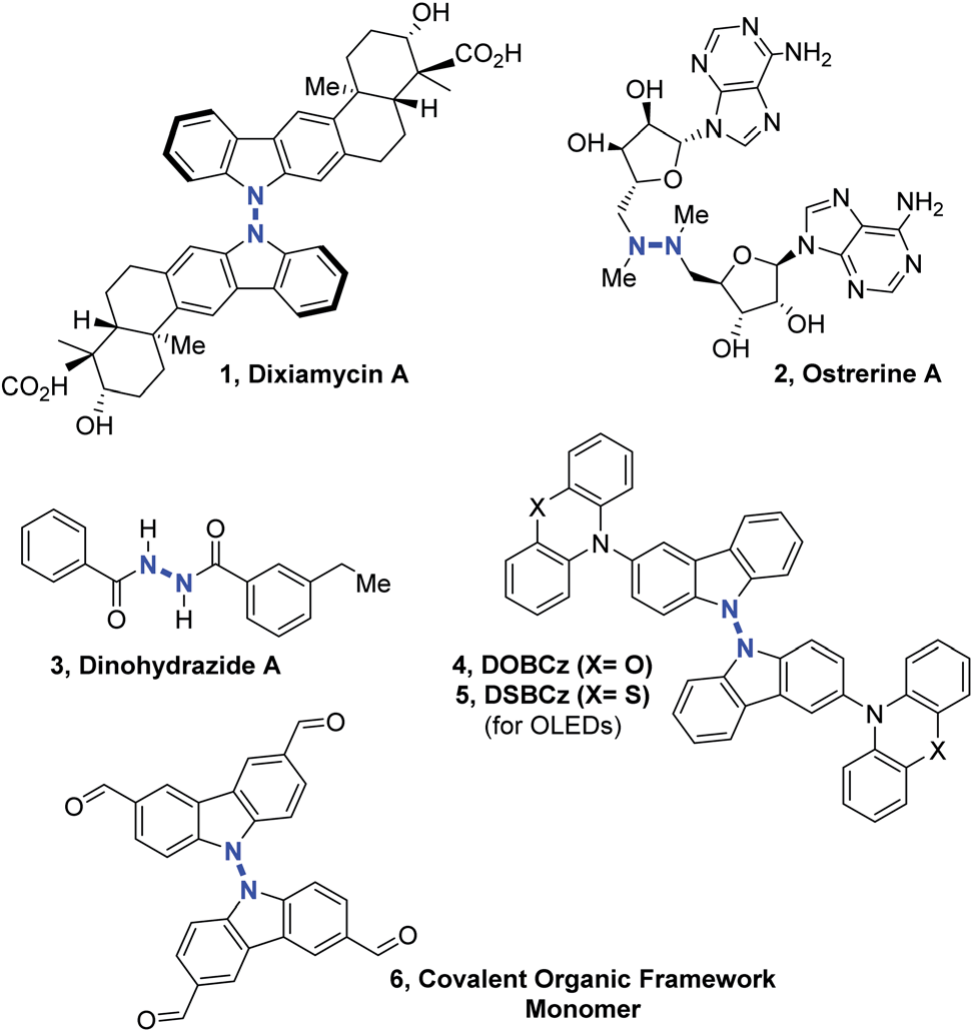
Examples of compounds potentially accessible by intermolecular N–N bond formation.

Nitrogen–nitrogen bond containing molecules are ubiquitously present in natural products^1^ and are increasingly used for applications in the field of organic materials such as organic light-emitting diodes (OLEDs)^2^ or covalent organic frameworks (COFs).^3^ In order to synthesize these relevant compounds (Scheme 1), it is necessary to find useful and effective methodologies. In the past few years, some intramolecular N–N bond forming methods became powerful tools to access different heterocycles containing the N–N motif, by cleavage of N–O, N–C, N–H or even N–N bonds.^4^ However, intermolecular N–N bond formation still remains a mostly unaddressed challenge despite the importance of scaffolds such as linked heterocycles, hydrazines or hydrazides in different fields of chemistry (Scheme 1).

In terms of methodology, there are essentially four retrosynthetic approaches possible for accessing the most challenging non-cyclic non-symmetrical N–N bonds. (1) The first and perhaps the simplest one is to start from the preformed N–N hydrazine motif itself and functionalize stepwise its up to four N-positions. This indirect solution will not be discussed here. (2) The second approach would be utilizing organometallic reagents and pre-functionalization at the N-atoms in order to promote a classical transition metal catalyzed cross-coupling reaction, which would lead to the formation of the desired N–N bond, for example in the frame of a final N–N reductive elimination step. However, the relatively high electronegativity of the N-atom makes this scenario very challenging. (3) The third option is to pre-install a leaving group on only one of the two N-coupling partners, so as to render it electrophilic.

**Scheme 2:**
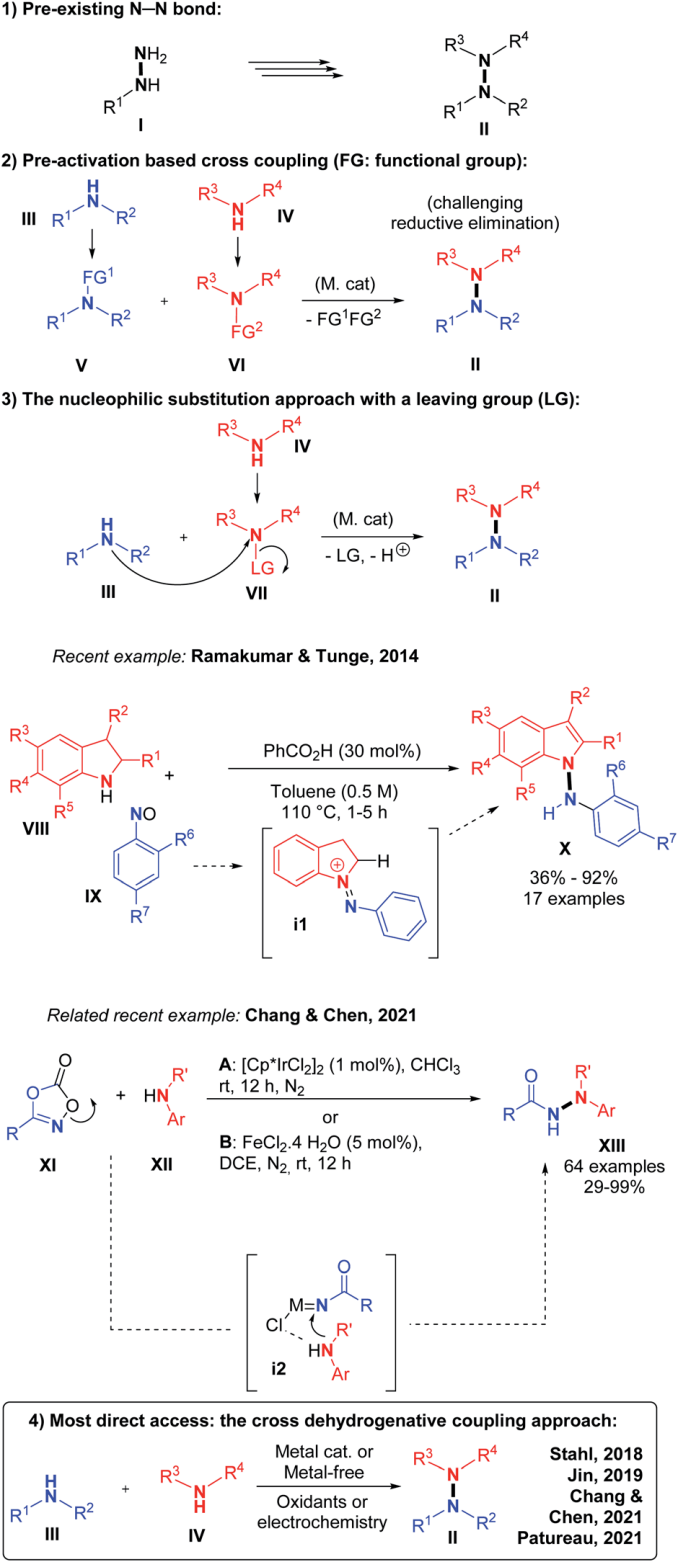
The N–N cross-dehydrogenative coupling approach.

This is equivalent to pre-oxidizing only one of the two coupling partners. Because N–H functional groups are often inherently nucleophilic due to their electronic lone pair as well as their relative softness, this third option is particularly adapted for the synthesis of intermolecular hetero N–N bonds. Indeed, side reactions such as N–N homo coupling reactions are thereby avoided due to the philicity control of the (hetero) N–N bond forming nucleophilic substitution process. In 2014, for example, Ramakumar and Tunge reported the N–N coupling of indoline with *N*-oxides.^5^ Similarly, Chang, Chen and co-authors recently demonstrated the power of such an approach with an elegant iridium catalyzed method, which proceeds through a nitrene intermediate.^6^ (4) The fourth strategy and also the most direct one, consists in performing an intermolecular hetero N–N bond formation by means of cross-dehydrogenative coupling (CDC, Scheme 2).^7^

CDCs are among the most sustainable coupling methods possible because they entirely avoid prefunctionalization of either coupling partners. They are therefore very atom- and step-efficient. However, N–N bond forming CDCs are associated with considerable challenges. Firstly, the high N-electronegativity implies the use of relatively strong oxidants in order to make the reaction thermodynamically feasible. These can in turn cause undesired side reactions, such as dehydrogenative C–N bond formation,^8^ which will necessarily limit the substrate scope of such methods. Secondly, the concept of CDC is limited by the often very favorable homo-coupling background processes. N–N bond forming CDCs are particularly susceptible to homo-coupling, as we shall see hereafter. Indeed, the most electron rich N-coupling partner tends to oxidize first, thereby becoming electrophilic and hence reacting with the most nucleophilic species present, usually another not yet oxidized equivalent of itself. In extreme cases, wherein one coupling partner is much more electron rich than the other, the oxidizing conversion of the latter will only begin when the former is essentially consumed, leading mostly to a mixture of two different homo-coupled products. CDC methods, including N–N bond forming CDCs, must therefore be designed to allow interception of the first oxidized coupling partner with the second. While this may seem challenging, the nature and structure of the oxidants, solvents, and catalysts can be tuned to enable this hetero interception process because these have per design a very intimate relationship with the substrates. This review will focus on the most recent developments in the area of intermolecular N–N bond forming CDCs, wherein the above mentioned challenges could be solved. Intramolecular examples will therefore not be considered.^4^

## 2. Dehydrogenative N–N homocoupling

**Scheme 3:**
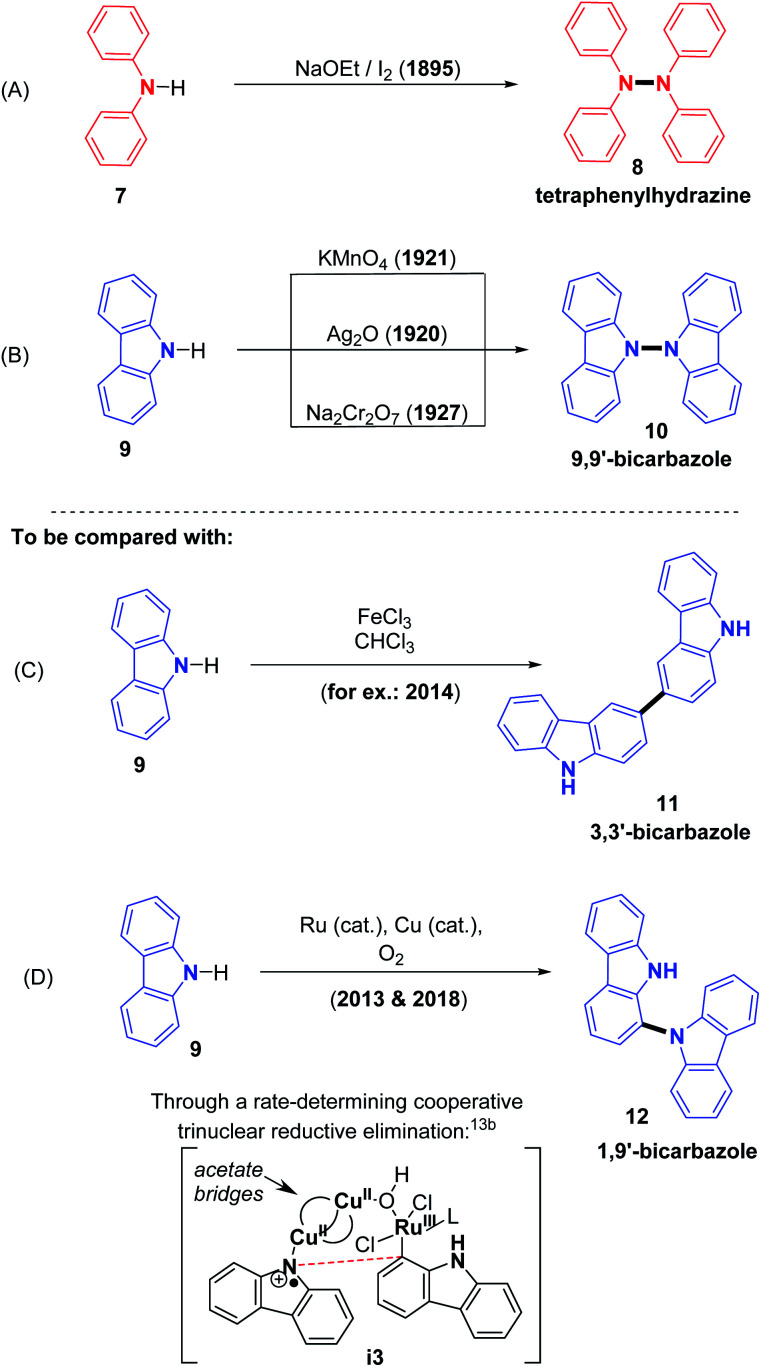
Seminal work concerning the oxidation of secondary amines and decisive impact of the oxidizing method.

The story of N–N bond forming CDCs begins at the very end of the 19th century, with the discovery of the synthesis of tetraphenylhydrazines from the direct oxidation of diphenylamine, described by Chattaway in 1895 using a strong base: sodium ethoxide combined with iodine (Scheme 3A).^9^ This discovery is particularly interesting in its historical context because it comes after the 19th century studies by Perkin on mauveine and related purple dyes, which are also obtained from anilines under oxidizing conditions, however through cross-dehydrogenative C–N oligomerization processes.^10^ Thus, it was already clear by then that the oxidizing method strongly impacts the fate of the substrates in terms of C–N *versus* C–C, or N–N dehydrogenative coupling. Similarly, various early methods were reported for the preparation of analogously structured 9,9′-bicarbazoles, utilizing stoichiometric oxidants such as Ag_2_O, KMnO_4_ and Na_2_Cr_2_O_7_, at the beginning of 20th century (Scheme 3B).^11^ The case of carbazole is particularly interesting because diverse oxidizing methods have been documented to selectively allow N–N (Scheme 3B),^11^ C3–C3 (Scheme 3C),^12^ and even C1–N (Scheme 3D)^13^ dehydrogenative bond formation, illustrating the decisive importance of the oxidizing system, and the absence or presence of catalysts. In the latter case (Scheme 3D), the unusual and highly selective C1–N bond formation process would be the result of a trinuclear bridged reductive elimination step, which would also be the rate limiting step of the reaction. This step is moreover in agreement with experimental kinetic investigations, displaying an optimal initial rate obtained with a 2 : 1 copper to ruthenium catalytic ratio, as well as with computational investigations.^13*b*^ Further on in this section, we will mainly describe the synthesis of di- and tetra-phenylhydrazines as well as 9,9′-bicarbazoles with dehydrogenative homocoupling reactions, utilizing diverse sets of reaction conditions, metal/oxidant, metal-free/oxidant and electrochemical systems.

### 2.1. Metal-catalyzed dehydrogenative N–N bond formation

**Scheme 4:**
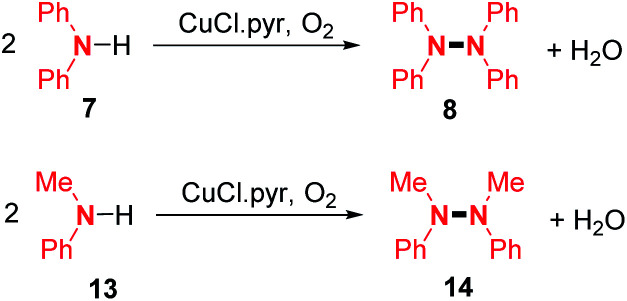
Cu-mediated N–N dehydrogenative coupling of secondary amines.

After several years of silence in this field, one of the first dehydrogenative N–N homocoupling reactions mediated by a copper salt was reported by Kajimoto and co-workers in 1982.^14^ In the presence of oxygen, they obtained the homocoupling products of diphenylamine **7** and *N*-methyl aniline **13** using an excess of CuCl in pyridine solution with 83% and 52% yields, respectively (Scheme 4).

**Scheme 5:**
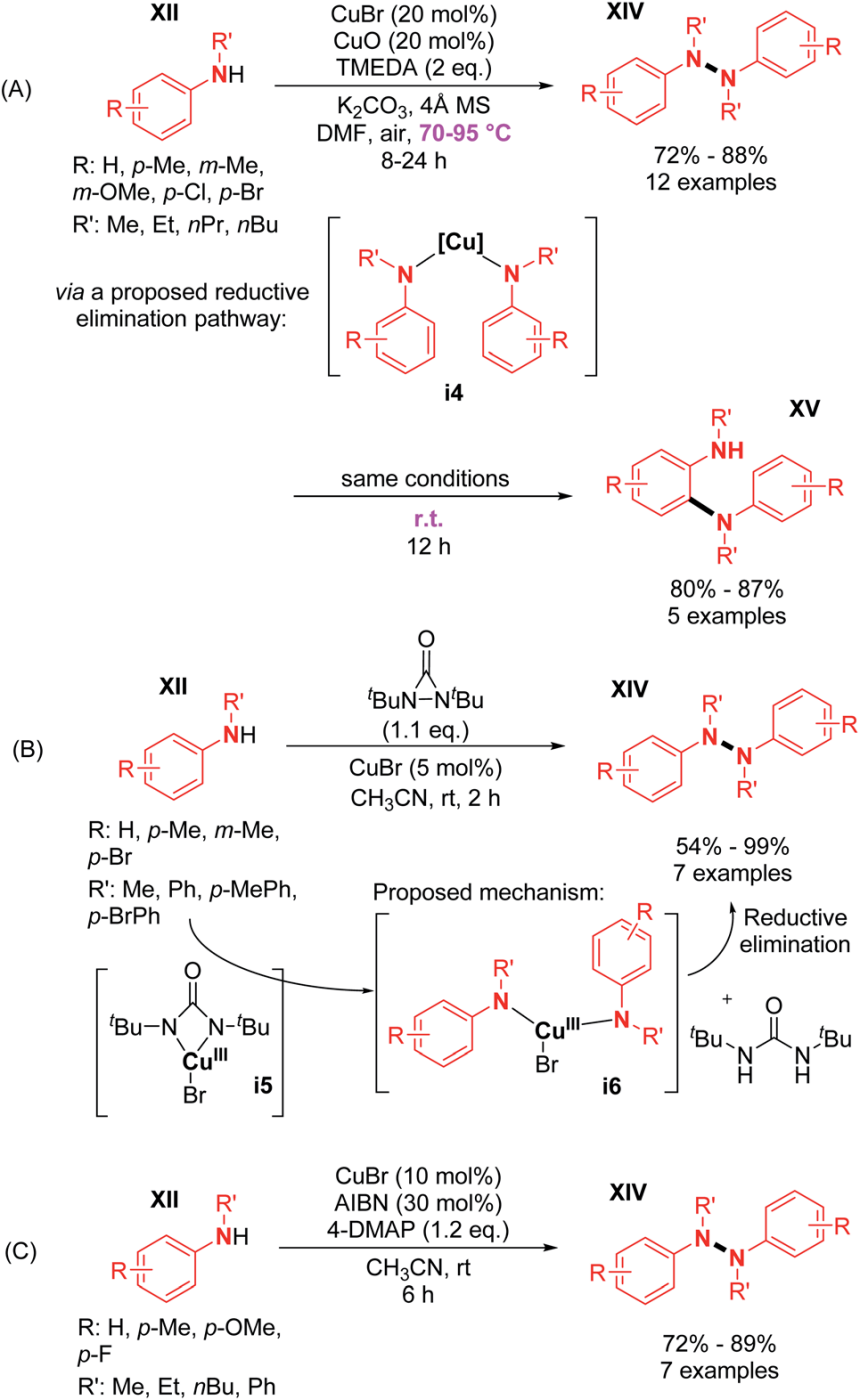
Cu-catalyzed N–N dehydrogenative coupling of secondary amines.

The authors suggested the formation of an amino radical in the coordination sphere of the copper-complex, which would attack another secondary amine and thus provides the desired N–N coupled product. In the years and decades thereafter, several research groups have developed related methods for the synthesis of di- and tetra-phenylhydrazines using copper salts as catalysts under various oxidizing conditions (Scheme 5). For example, Huang and co-workers described the synthesis of diphenylhydrazines from *N*-alkyl anilines in good yields (Scheme 5A).^15^

**Scheme 6:**
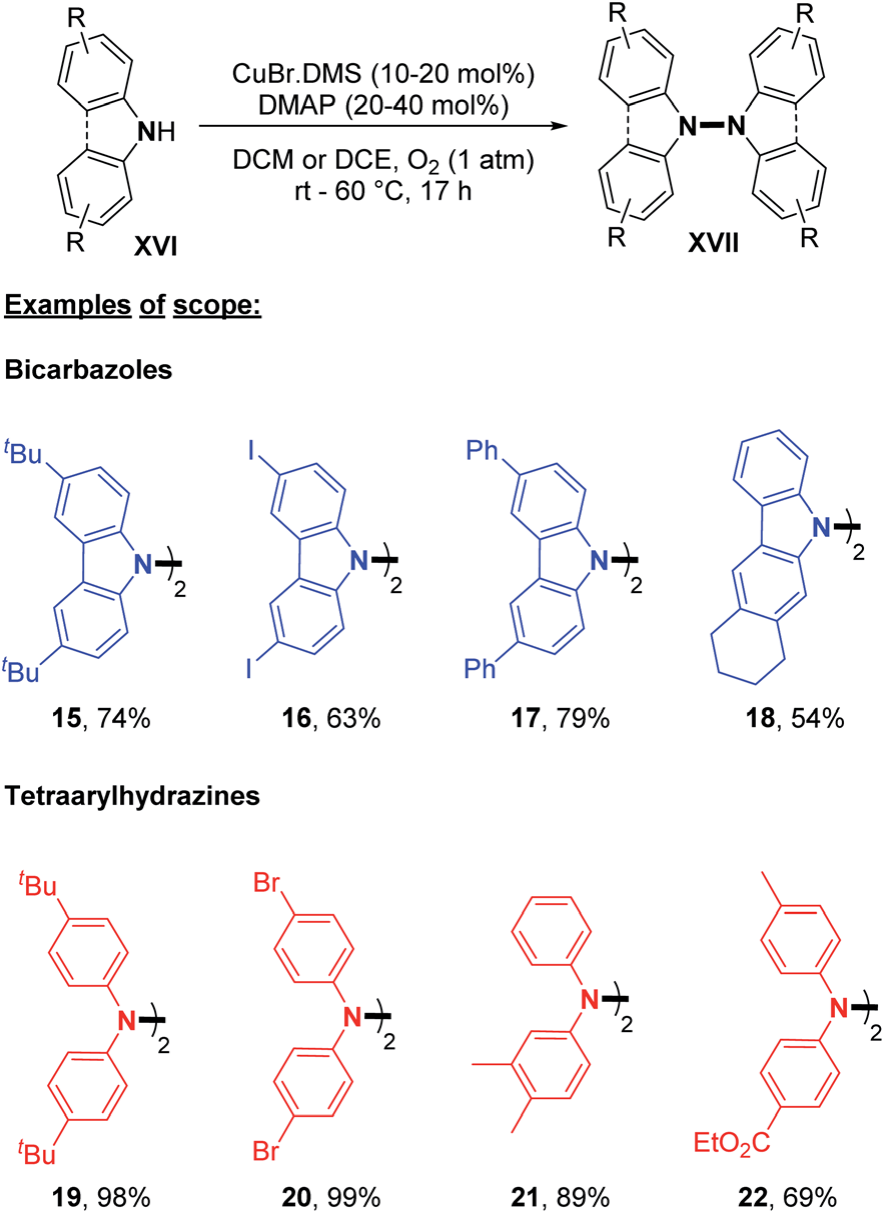
Formation of di- and tetra-phenylhydrazines.

The major advantage of this strategy is the use of simple air as an oxidant instead of stoichiometric chemical oxidants. A possible mechanism described by the authors is (i) the coordination of the peroxo-dicopper(ii) complex formed *in situ* by oxidation, (ii) nucleophilic attack by *N*-alkynylamine, and (iii) reductive elimination. The presence of a Cu(ii) oxide additive was moreover speculated by the authors to facilitate oxygen activation and subsequent N–H activation at the copper center. Amazingly, the same reaction conditions at room temperature led to the formation of *o*-semidines *via* a dehydrogenative C–N bond formation (5 examples, 80–87%). This temperature controlled orthogonality illustrates again the central importance of the fine-tuning of the oxidizing method with respect to C–C, C–N, or N–N dehydrogenative bond formation. A few months later, in 2013, Shi and Zhu developed a novel dehydrogenative homocoupling of secondary anilines using di-*tert*-butyldiaziridinone as an oxidant to access various functionalized tetraphenylhydrazines in moderate to good yields (Scheme 5B).^16^ The authors proposed a nitrogen radical mediated hydrazine dimerization. This radical is obtained by the reductive cleavage of the N–N bond of di-*tert*-butyldiaziridinone by CuBr allowing the formation of Cu(ii) nitrogen radicals, which would react with secondary anilines. This methodology was also applied to primary anilines to produce substituted azobenzenes. Additionally, Reddy and co-workers reported a copper-catalyzed dehydrogenative homocoupling for the preparation of diphenylamines and azobenzenes (respectively from secondary and primary anilines) in good yields utilizing AIBN in sub-stoichiometric amounts (Scheme 5C).^17^ Recently, Stahl and co-workers described an efficient copper-catalyzed dehydrogenative homocoupling of carbazoles under mild conditions using O_2_ as the oxidant.^18^ They also applied this methodology for the homocoupling of diarylamines. The most intriguing aspect of this contribution was the identification of the first hetero selective N–N bond forming CDC product between carbazole and diarylamine (see Section 3). In order to first optimize the dehydrogenative homocoupling of carbazole, the authors utilized 3,6-di(*tert*-butyl)carbazole as a model substrate. With the optimized conditions in hand, they expanded the scope to carbazoles bearing both electron-withdrawing and electron-donating groups at the 3- and 6-positions (Scheme 6; *cf.***15**, **16**, and **17**) as well as to relevant unsymmetrical carbazoles (Scheme 6; *cf.***18**), representing a structural analogue of dixiamycin A (Scheme 1).

**Scheme 7:**
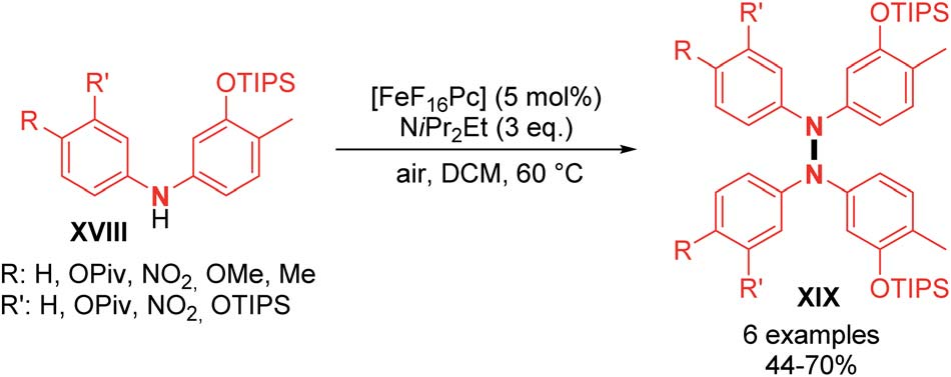
Iron-catalyzed oxidative N–N homocoupling of diarylamines.

Dehydrogenative N–N dimerization of diarylamines was also achieved with high yields under similar conditions. Near-quantitative yields were obtained with diarylamines bearing both electron-withdrawing and electron-donating groups (Scheme 6; *cf.***19**, **20**, **21**, and **22**). Importantly, tetraarylhydrazine **19** was recently utilized as an efficient organocatalyst for the electrochemical synthesis of imidazo-fused N-heteroaromatic compounds.^19^ A good yield was also obtained for unsymmetrical diarylamine but required an increase in temperature to 60 °C (Scheme 6; *cf.***22**). Besides copper, few other metals were utilized to catalyze dehydrogenative N–N homocoupling reactions. Knölker and co-workers developed an iron-catalyzed oxidative homocoupling of diarylamines^20^ using phthalocyanines, an important class of ligand in catalysis,^21^ and Hünig's base as an additive. They described the formation of six tetraarylhydrazines in moderate to good yields (Scheme 7). In addition, diarylamines **XVIII** also allowed the synthesis of 2,2′-bis(arylamino)biaryls by C–C bond formation and 5,6-dihydrobenzo[*c*]cinnolines by C–C and N–N bond formations using the same catalyst. The selectivity only depends on the choice of the additive (respectively methanesulfonic acid and acetic acid).

**Scheme 8:**
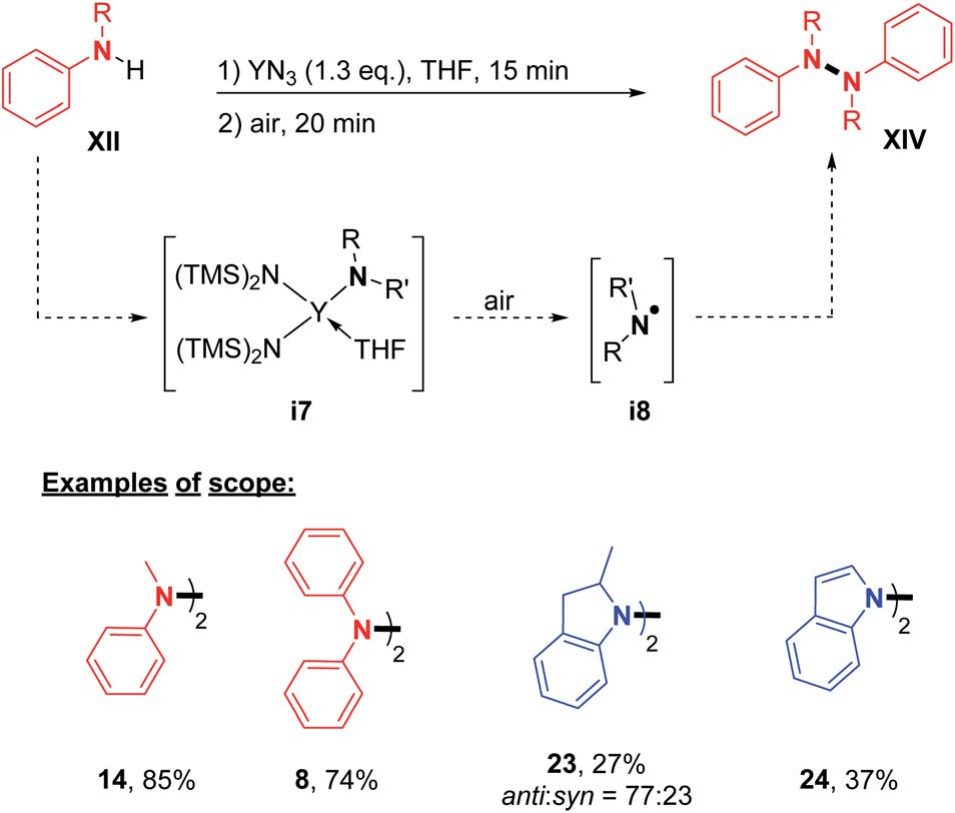
Oxidative homocoupling reaction of secondary amines using rare-earth-metals.

Finally, Wang, Zhang, and co-workers reported an efficient synthesis of hydrazine derivatives utilizing a rare-earth-metal. YN_3_ was used in stoichiometric amounts to provide the desired N–N product in moderate to good yields (Scheme 8).^22^ In order to confirm the reaction mechanism, the authors isolated the N–Y complex **i7** formed during the first step, and proved the formation of the N-radical intermediate **i8** by EPR, leading to the N–N homocoupling product. With this method in hand, they described the synthesis of di- and tetra-phenylhydrazines (Scheme 8; *cf.***14** and **8**) and also the unique cases of the formation of 1,1′-bi(2-methyl)indoline and 1,1′-biindole (Scheme 6; *cf.***23**, and **24**).

### 2.2. Metal-free dehydrogenative N–N bond formation

**Scheme 9:**
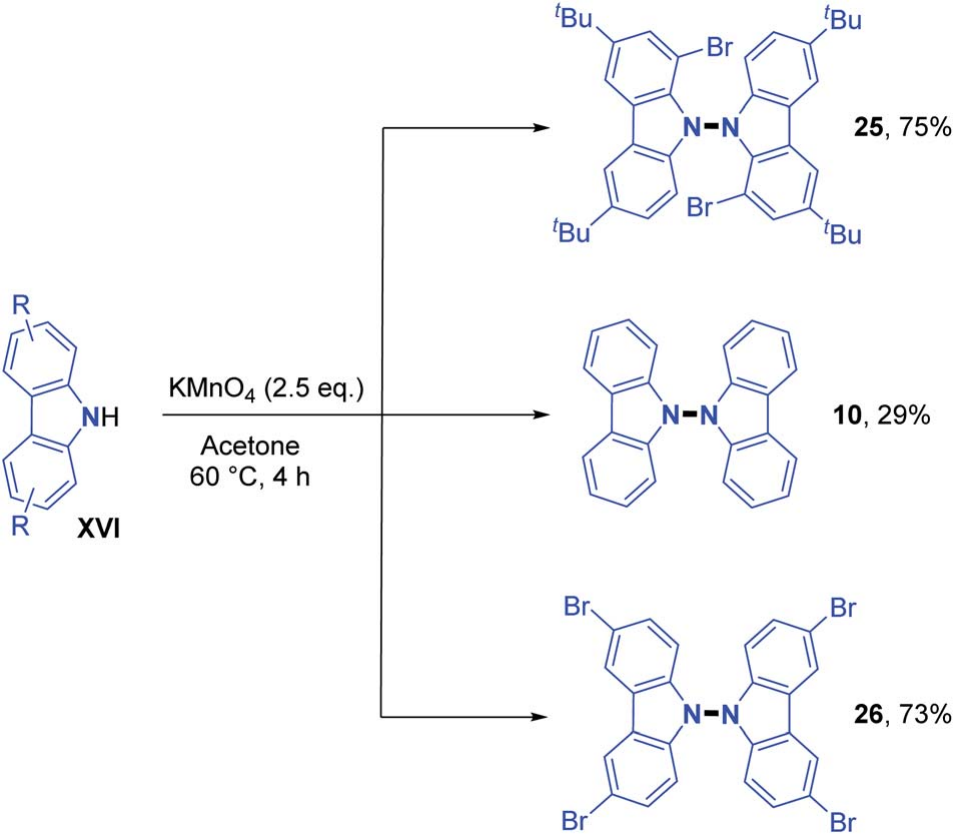
Synthesis of 9,9′-bicarbazoles with KMnO_4_ as the oxidant.

The direct handling of strong oxidants also allows in some cases intermolecular dehydrogenative N–N bond formation under metal-free conditions, as previously described by Tucker and Perkin. In recent times, the 9,9′-bicarbazole motif was obtained using potassium permanganate as an oxidant.^23^ In addition, Higashibayashi's and Chen's group synthesized the bromo-substituted 9,9′-bicarbazoles **25** and **26** in good yields (around 75%) and standard 9,9′-bicarbazole **10** in a moderate 29% yield (Scheme 9).^23^ This method seems more effective with electron rich carbazoles.

**Scheme 10:**
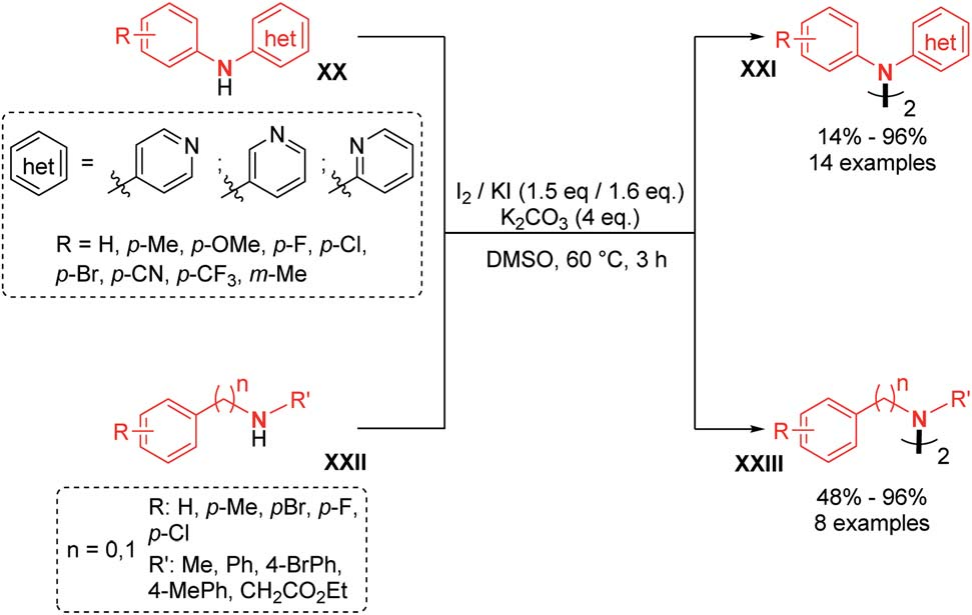
I_2_/KI-mediated dehydrogenative N–N bond formation.

**Scheme 11:**
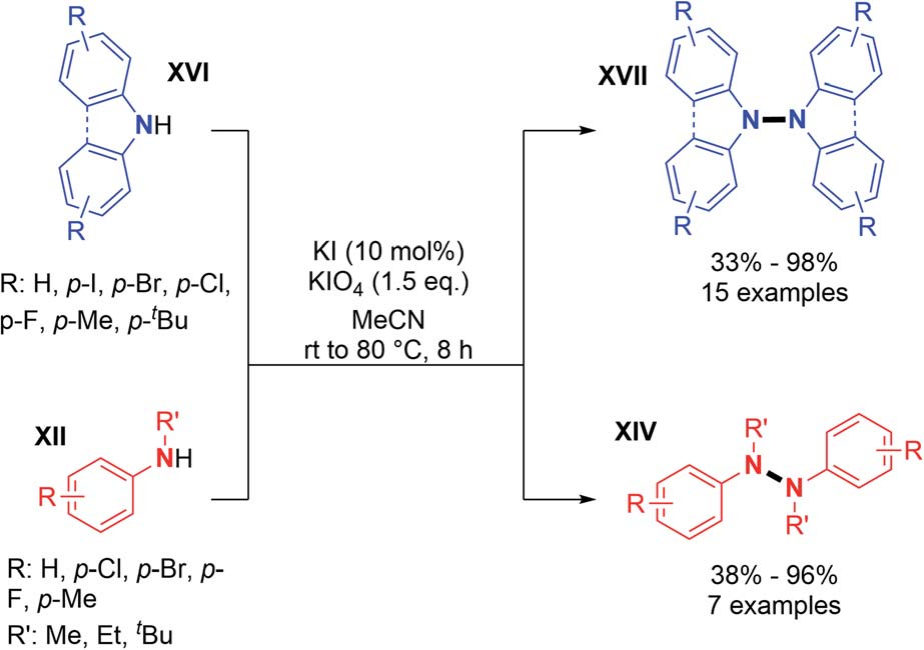
KI/KIO_4_-mediated dehydrogenative N–N bond formation.

Recently, two groups utilized molecular iodine as the sole oxidant for the dehydrogenative N–N bond formation of secondary amines. Yu, Chang and co-workers described the synthesis of new varieties of hydrazines from *N*-aryl aminopyridines, engaging I_2_/KI as an oxidizing system.^24^ This method was extended to the oxidative dimerization of diarylamines and *N*-alkyl anilines with good yields (Scheme 10). The authors suggested that the presence of KI allows a better efficiency of the reaction *via* the formation of a KI_3_ intermediate. Without KI, the conversion was slow and the desired product was formed in decreased yield. Mechanistic investigations suggested the formation of an *N*-iodo intermediate. The same year, Yin and Jin also reported a transition-metal-free dehydrogenative N–N bond formation method utilizing a KI/KIO_4_ system for the formation of di- and tetra-phenylhydrazines and 9,9-bicarbazoles (Scheme 11).^25^ Moreover, under these metal-free conditions, they demonstrated the feasibility of the dehydrogenative N–N hetero-coupling between arylamines and carbazoles (see Section 3). Generally, good yields were obtained, mostly for the electron-rich substrates.

Recently, the renaissance of electrochemistry in the field of organic synthesis^26^ has allowed the development of successful electrochemical intermolecular N–N bond constructions.

**Scheme 12:**
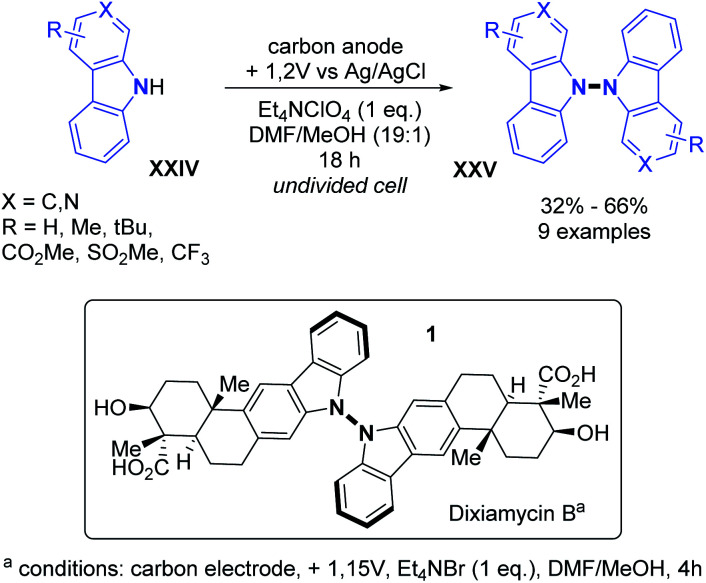
Electrochemical dehydrogenative N–N dimerization of carbazoles and carbolines, route to the total synthesis of dixiamycin B.

Indeed, anodic oxidation can generate N-centred radicals from amines.^27^ The first example based on electrochemical oxidation was reported by Baran and co-workers for the total synthesis of dixiamycin B in 2014.^28^ By employing carbon electrodes, the N–N oxidative dimerization was evaluated for substituted carbazoles and carbolines (Scheme 12). Generally good yields and good functional group tolerance such as ester, alkyl and sulfone were observed. Utilizing these conditions, the first total synthesis of dixiamycin B was obtained by the N–N oxidative dimerization of xiamycin A in the final step, with a yield of 28%.

**Scheme 13:**
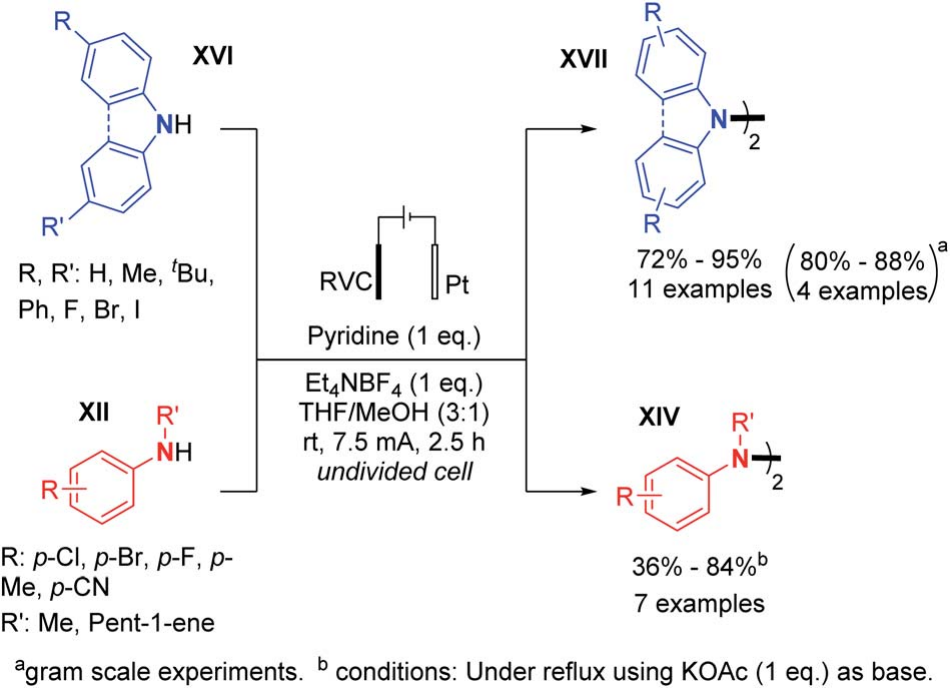
Other electrochemical synthesis of tetrasubstituted hydrazines by dehydrogenative N–N bond formation.

In 2019, Xu and co-workers generalized the dehydrogenative N–N dimerization of secondary amines using electrochemistry.^29^ Employing a base, in contrast to Baran's conditions, they described the synthesis of substituted di- and tetra-phenylhydrazines and 9,9-bicarbazoles in very good yields and also performed gram scale synthesis (Scheme 13). A plausible reaction mechanism was hypothesized: (i) the secondary amine would first be oxidized on the anode to form a radical cation; (ii) then the radical cation would be deprotonated with the base; (iii) the aminyl radical would be generated, thus allowing homodimerization. According to the authors, the oxidative dimerization reaction is probably self-catalyzed, and the hydrazine product would serve as a redox catalyst for the anodic generation of radical cations.

**Scheme 14:**
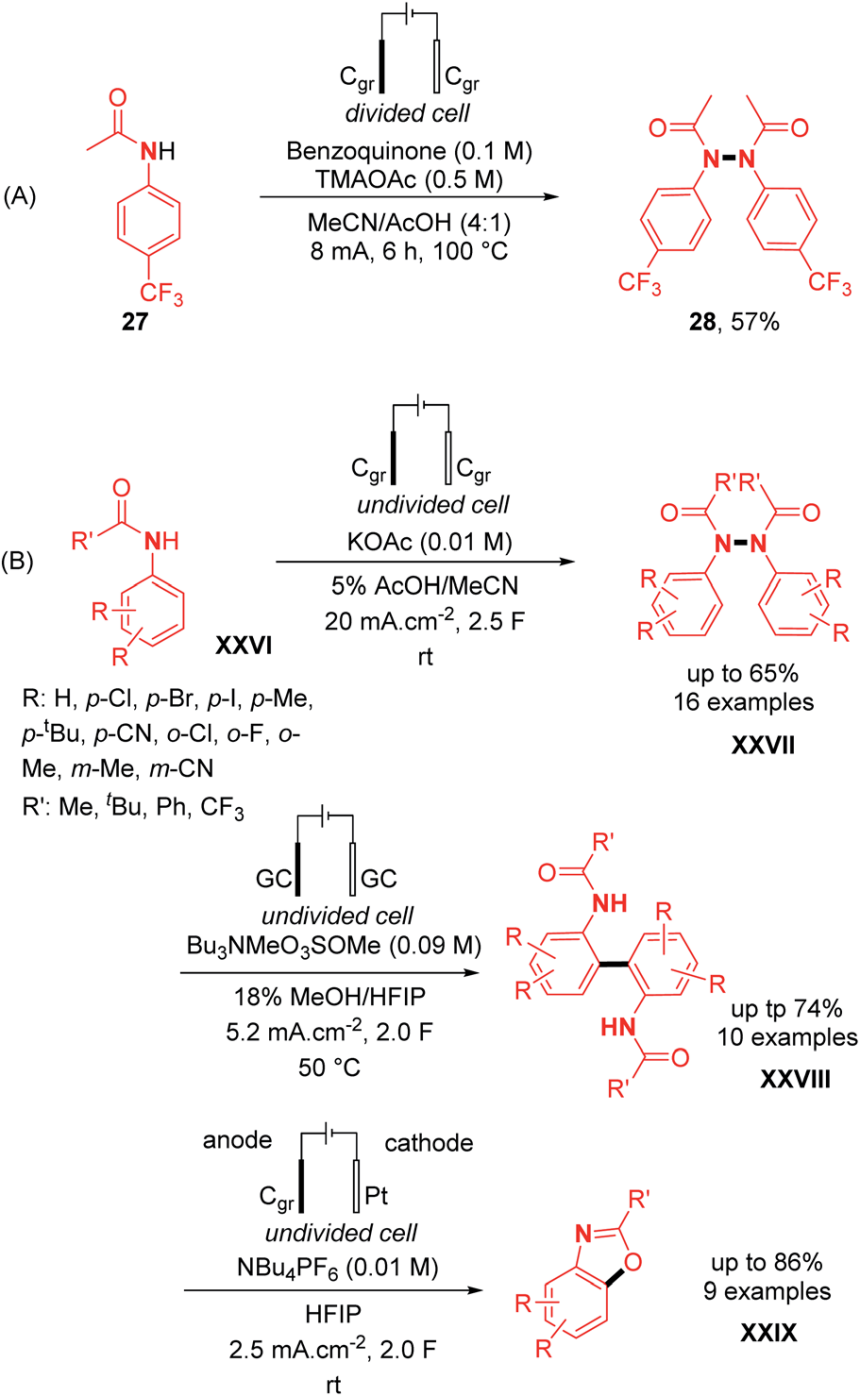
Dehydrogenative anodic oxidation of anilides for N–N bond formation.

Engaging anilides as coupling partners, a new variety of *N*,*N*′-diphenylhydrazine was also reported by electrochemical oxidation. Interestingly, the first example of N–N dimerization of anilides was discovered by Sanford and co-workers as a side-product of a Pd-catalyzed electrochemical acetoxylation of C–H bonds.^30^ From acetanilide **27**, the oxidative N–N bond formation occurred to afford product **28** with a yield of 57% under the standard reaction conditions (Scheme 14A). Nevertheless, the use of a divided electrochemical cell at 100 °C remains a drawback of this method. In contrast, utilizing an undivided electrochemical cell moreover under mild conditions, Liermann, Waldvogel and co-workers explored the dehydrogenative N–N homocoupling of anilides (Scheme 14B).^31^

The authors also optimized the reaction with acetanilide **27** and the N–N dehydrogenative homocoupling product **28** was obtained with yields comprised between 63% and 66% depending on the scale. Concerning the scope of the reaction, several diversely substituted acetanilide N–N dimers were obtained in good to moderate yields (up to 66%) (Scheme 14B). In order to investigate the orthogonality of their method, halide and cyano derivatives were also used and found compatible. Moreover, in the same article, modifying the electrochemical parameters afforded other C–C and C–O dehydrogenative bond forming products instead of N–N, demonstrating once more the versatility of the concept of dehydrogenative N–N bond formation (Scheme 14B).

**Scheme 15:**
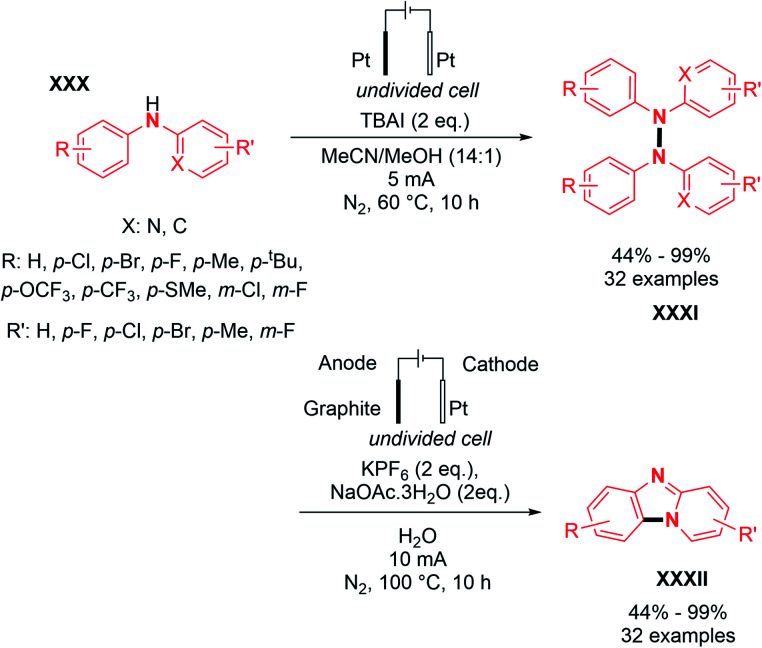
Electrochemical dehydrogenative N–N bond formation of *N*-aryl aminopyridines.

Finally, based on the captodative effect (stabilization of a radical through both donating and withdrawing substituents), Chen and co-workers reported the N–N oxidative homocoupling with *N*-aryl aminopyridines under electrochemical conditions.^32^ Using an electrolysis set-up composed of platinum electrodes in an undivided cell, they proposed the preparation of hydrazines with a broad scope including electron rich groups such as alkyl, electron deficient groups such as halogens and trifluoromethyl moieties in good yields, up to 99% (Scheme 15).

**Scheme 16:**
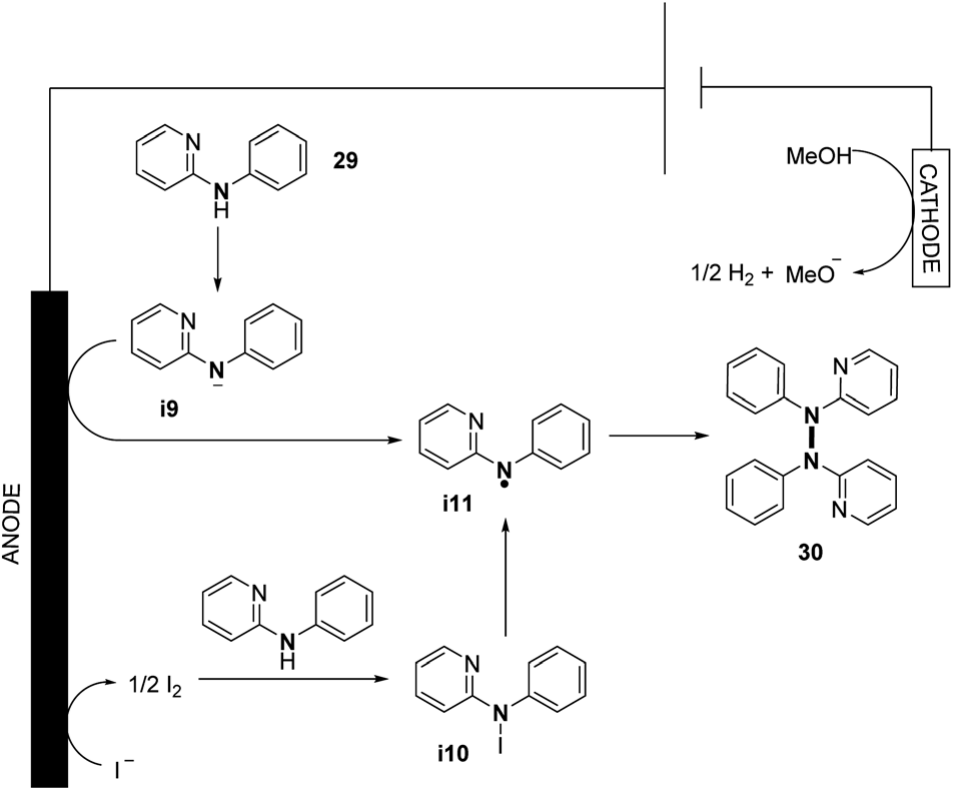
Proposed mechanism of the electrochemical oxidative N–N bond formation of *N*-aryl aminopyridines.

They also explored the possibilities of hetero-coupling reactions under their conditions (see Section 3). Interestingly, by changing the electrochemical parameters and additives of the reaction, an intermolecular C–N cyclization was also explored. The authors performed preliminary mechanistic investigations with a series of control experiments, cyclic voltammetry, and DFT study. They conjectured two possible pathways for the formation of N-centered radical **i11**, allowing N–N bond formation (Scheme 16). The first one would be the direct oxidation of the nitrogen anion intermediate **i9** formed by the deprotonation of *N*-aryl aminopyridine. The second one would be *via* the formation of intermediate **i10** from the anodic oxidation of the iodide anion, followed by the homolytic cleavage of the N–I bond. According to the authors, good solubility of the substrate and high concentration of the N-centered radical **i11** promote N–N coupling.

## 3. Dehydrogenative N–N heterocoupling

**Scheme 17:**
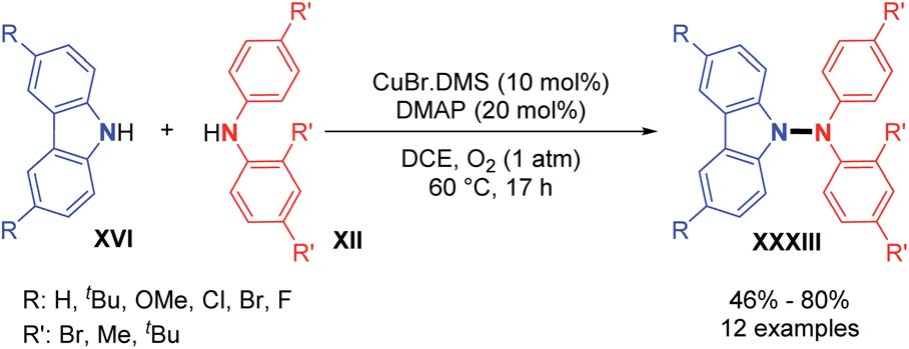
Cu-catalyzed dehydrogenative hetero N–N bond formation.

Utilizing the copper-catalyzed system previously described for the dehydrogenative N–N homocoupling reaction of carbazoles and diarylamines (Scheme 6), Stahl and co-workers also showed the possibility of dehydrogenative hetero-coupling reactions between carbazoles and diarylamines.^18^ Preliminary experiments showed that a small excess of carbazole (1.5 equivalents) led to the formation of the N1–N2 cross-coupled product in high yield, as opposed to equimolar amounts of carbazole and diarylamine. From these observations, they explored the scope of the cross-coupling reaction. Carbazoles reacted well in hetero-coupling reactions with diarylamines bearing electron-withdrawing groups (–Br) or electron-donating groups (–*t*Bu). When a Br-substituted diarylamine was employed as a coupling partner, both electron rich and poor carbazoles produced the cross-coupled product in good yields. However, the homocoupling of carbazoles remains a competent side reaction in this method (Scheme 17).

**Scheme 18:**
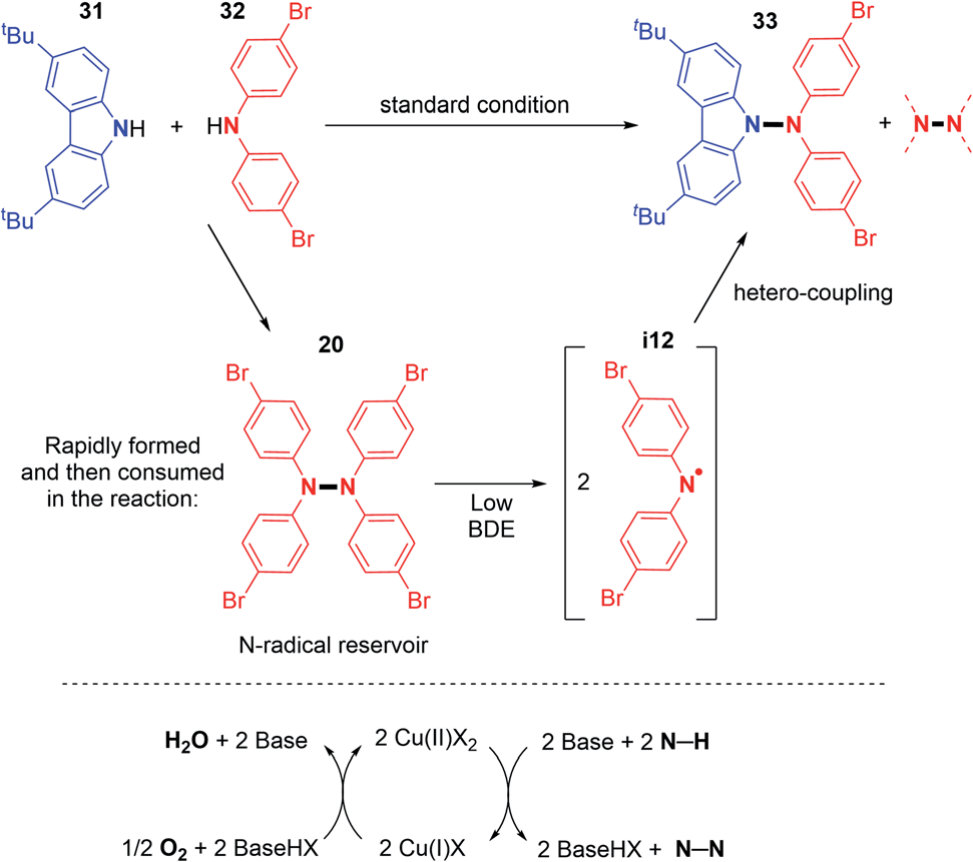
Origin of the N1–N2 hetero-coupling product and proposed oxidative mechanism.

In order to understand the origin of the heteroselectivity, the authors conducted ^1^H NMR experiments during the reaction. They noticed that the diarylamine homocoupling product is rapidly generated and then disappears with the formation of the cross-coupling product (Scheme 18).

EPR experiments suggested N-centered radical formation under reaction conditions from tetraarylhydrazines. Indeed, the homolytic cleavage of the N–N bond was envisaged due to the weakness of the bond dissociation energy of diarylamines (BDE = 23.5 kcal mol^−1^ for Ph_2_N–NPh_2_).^33^ This would generate the N-centered radical that readily reacts with carbazoles and forms the desired N–N hetero-coupling product (Scheme 18). In order to better understand this reaction, the same group recently reported new mechanistic insights.^34^ In particular, the authors showed that under such reaction conditions, typically first order kinetics are observed for the O_2_ gas, while second order kinetics are observed for the copper salt, indicating that the copper catalysed activation of O_2_ is a key step in this reaction (Scheme 18). In contrast, the N–H substrate seems to display a negative kinetic order. Thus, it inhibits the reaction, possibly through saturation of the coordination sites at the copper centre.

**Scheme 19:**
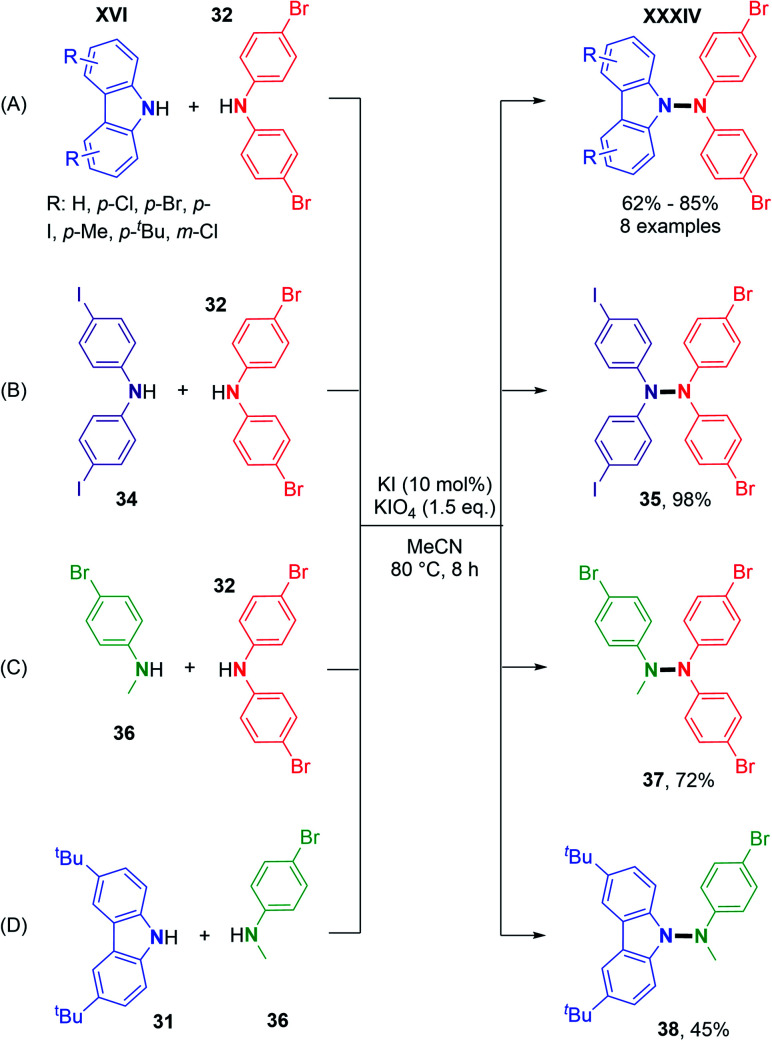
Metal-free dehydrogenative hetero N–N cross-coupling reactions.

Using metal-free conditions as previously described for the oxidative N–N homocoupling reaction, Jin and co-workers also explored the hetero-coupling scenario.^25^ In contrast to Stahl's conditions, the hetero-coupling reaction performed well with equimolar amounts of the two coupling partners, and no homocoupling products seem to have been observed. They described the cross-coupling reaction between carbazoles bearing electron-withdrawing groups and electron-donating groups and bis(4-bromophenyl)amine in good yields (Scheme 19A). They also expanded the scope to heterocouplings between different diarylamines in excellent yields (Scheme 19B). Interestingly, products **37** and **38** showed the possibility of utilizing *N*-alkyl anilines as coupling partners with carbazoles or diarylamines (Scheme 19C and D).

**Scheme 20:**
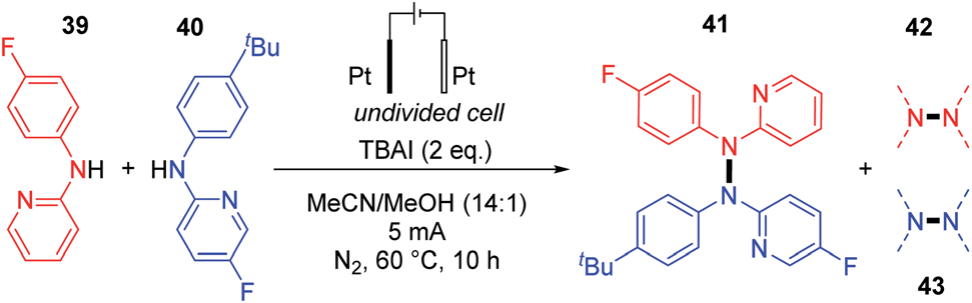
Electrochemical oxidative hetero N–N coupling reaction.

Based on similar control experiments to Stahl's report, the authors explained the heteroselectivity in the coupling product by the N–N bond homolytic cleavage of tetraarylhydrazine, generating the N-centered radicals. Finally, employing their electrochemical conditions, Chen and co-workers also showed the feasibility of dehydrogenative hetero N–N coupling reactions between different *N*-aryl aminopyridines.^32^ Their report suggests that the reaction efficiency vastly depended on the relative stoichiometry of the two coupling partners (Scheme 20). Using equimolar amounts of substrates led to an almost statistical distribution of homo- and hetero-coupled products. In an attempt to bypass homocoupling, a large excess (5 equivalents) of one of the two coupling partners was used, which significantly improved the formation of the hetero-coupled product in 75% yield.

**Scheme 21:**
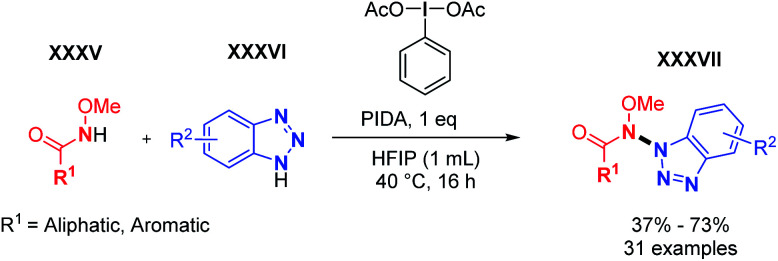
Heterodehydrogenative N–N coupling of amides and benzotriazoles.

Realising the significant scope limitations of hetero N–N coupling reactions as illustrated by previous studies, we sought out to expand the dehydrogenative hetero N–N bond forming toolbox. Thus, in 2021, we developed the cross-dehydrogenative N–N coupling of aromatic and aliphatic methoxyamides with benzotriazoles (Scheme 21).^35^ Utilizing a simple hypervalent oxidant (diacetoxyiodobenzene, PIDA), the N–N hetero dehydrogenative coupling of methoxyamides and benzotriazoles was achieved for the first time with excellent heteroselectivity. Both aliphatic and aromatic methoxyamides yielded the corresponding N–N product in good to moderate yields. The reaction also showed very good functional group tolerance to both coupling partners. The *N*-methoxy functional group seems to play a decisive role in the observed reactivity. Indeed, it likely stabilizes either the N-radical or alternatively the N-cation through the mesomer donor effect. The reaction does not proceed in its absence. Moreover, the *N*-methoxy group is trivial and easily installed, while benzamides are among the most important scaffolds in organic chemistry. Further attempts to expand the scope of N–N bond forming reactions are currently ongoing in our laboratory.

## 4. Conclusions

With the recent inclusion of certain amides^30,31^ into the concept of intermolecular heterodehydrogenative N–N bond formation,^35^ a certain frontier has been crossed. Indeed, the concept is no longer limited to carbazoles and structurally related diarylamines, which were the typical substrates for well over a century. This change of paradigm is likely to encourage further method development in this area in the coming months and years. In addition, some of the tools that enable dehydrogenative N–N bond formation have witnessed considerable advances over the last few years, such as electrochemical synthesis,^26^ photoredox catalysis,^36^ and hypervalent halide mediated, or catalyzed oxidative coupling reactions,^37^ to mention but a few key domains. Thus, an increase in relevant heterodehydrogenative N–N bond formation methods can be expected in the near future.

**Scheme 22:**
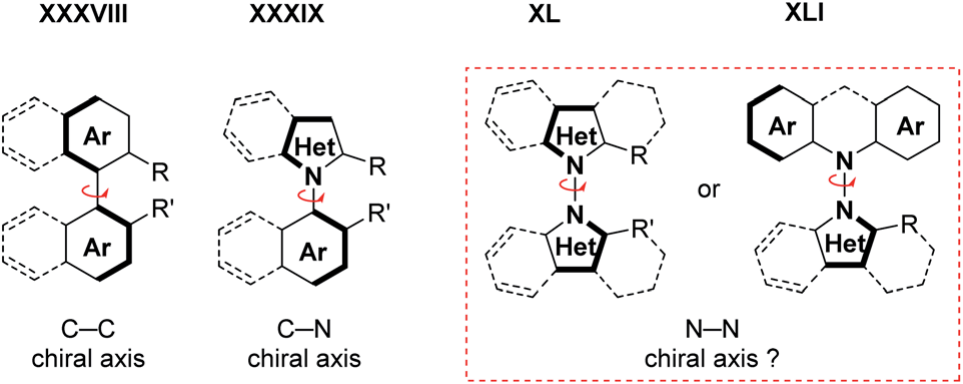
Towards atroposelective/enantioselective N–N bond formation.

Finally, the increasing availability of methods to access these scaffolds might eventually enable atroposelective (dehydrogenative) N–N bond forming methods, analogous to the currently emerging atroposelective C–C^38^ and C–N^39^ bond forming reactions (Scheme 22). The idea is certainly challenging,^40^ but will probably be successful soon in view of the recent progress in atroposelective coupling reactions.^38,39^ The general importance of the N-atom in organic chemistry suggests that these methods and their products will be uniquely useful.

## Author contributions

Writing – original draft: A. T. Writing – review & editing: A. T., P. Y. V. and F. W. P. Supervision: F. W. P.

## Conflicts of interest

There are no conflicts to declare.

## Supplementary Material
